# Gene expression and genetic control to cold tolerance during maize seed germination

**DOI:** 10.1186/s12870-020-02387-3

**Published:** 2020-04-29

**Authors:** Izabel Costa Silva Neta, Édila Vilela de Resende Von Pinho, Viviane Maria de Abreu, Danielle Rezende Vilela, Milena Christy Santos, Heloisa Oliveira dos Santos, Ricardo Augusto Diniz Cabral Ferreira, Renzo Garcia Von Pinho, Renato Coelho de Castro Vasconcellos

**Affiliations:** 1 Helix Sementes, Patos de Minas, Minas Gerais Brazil; 2 Department of Agriculture, Universidade Federal de Lavras, Lavras, Minas Gerais Brazil; 3 Bayer Cropscience, São Paulo, São Paulo Brazil; 4 Syngenta, Uberlândia, Minas Gerais Brazil

**Keywords:** *Zea mays*, Vigor, Heterosis, Maternal effect

## Abstract

**Background:**

The study of cold tolerance in maize seeds and seedlings through physiological quality assessments, as well as the genetic control associated with this trait, allows an early characterization of genotypes. Here we studied the genetic control for cold tolerance during the germination process in maize seeds and genes influenced by this stress.

**Results:**

Six maize lines were used, three classified as tolerant and three as susceptible to low germination temperature. A field was developed to produce the hybrid seeds, in a partial diallel scheme including the reciprocal crosses. For the expression analysis, seeds from two contrasting lines were used, as well as their hybrid combination and their reciprocal crosses, on dried and moistened seeds at 10 °C for 4 and 7 days. It was evaluated the catalase (CAT) and esterase (EST) enzymes, heat-resistant proteins and the genes Putative stearoyl-ACP desaturase (SAD), Ascorbate Peroxidase (APX), Superoxide Dismutase (SOD) and Mitogen Activated Protein Kinase (ZmMPK5). The estimated values ​​for heterosis, general and specific combining abilities and reciprocal maternal and non-maternal effects were carried out and it showed that there is heterosis for germination at low temperatures, also the non-additive genes were more important and there was a reciprocal effect.

**Conclusions:**

There is a greater expression of the CAT and EST enzymes in moistened seeds at seven days and there is less expression of heat-resistant proteins and the SAD gene at seven days of moistening. Also, there are variations in the expression of the APX, SOD and *ZmMPK5* genes in dried and moistened seeds, as well as among the genotypes studied.

## Background

The selection of maize cultivars with cold tolerance is fundamental to guarantee the germination, the establishment of seedlings and the productivity of grains under low temperatures. Under proper temperature conditions, maize seeds tend to germinate in three to four days. When the temperature is reduced to 15 °C the germination period can be up to 14 days, and as the temperature is reduced, the time required for the germination process increases [1]. Besides the lack of uniformity and the delay in the germination process, another problem associated with the occurrence of low temperatures is the fact that the seeds will stay longer in the soil exposed to deterioration and pest attack.

Increasing the tolerance of plants to environmental stresses is one of the most relevant goals of breeding programs and selection of superior genotypes should take into account the seed germination and seedling emergence phases. In some studies, the effect of heterosis on the cold tolerance characteristic has been observed.

The better performance of hybrids compared to parental lines corroborates with research results in which the regulation of cold tolerance involves additive and dominance effects [2, 3]. Thus, it is important to evaluate the tolerance to cold stress in seeds in the selection processes, when the best hybrid combinations are still being tested.

Adaptation to abiotic stress requires an increase in traits that are quantitatively inherited and highly influenced by the environment. These traits are mostly controlled by hundreds of genes and their interactions are often difficult to measure [4]. Many genes have been reported as of potential importance to increase cold tolerance during the germination process, however, only a limited amount of QTLs have been shown to have a real effect on this trait [5–8].

The best way to obtain cold-tolerant maize genotypes during the germination process is to know the genes related to this trait and to check whether or not there is an effect of heterosis and reciprocal effect. It is known that in maize breeding programs the end-use of the lines is for hybrid production, so it is important to identify the best genotypes and the best combinations. The study of cold tolerance in maize seeds and seedlings, through evaluations of the physiological quality of seeds, as well as the study of the genetic characteristics associated with this trait, allows an early characterization of the genotypes with tolerance to this adverse temperature condition.

In view of the above, the goal of this study was to study the genetic control for cold tolerance in germination and to evaluate the expression of genes associated with this trait in maize seeds.

## Results

The mean water content of the seeds at the time of the tests was 12.8 with a maximum variation of 1%.

Regarding the physiological quality, when the germination was carried out at 25 °C, there were no significant differences between the evaluated materials (Table 1). Thus, it can be affirmed that all analyzed materials, parent lines and hybrids, presented similar results when evaluated at 25 °C, which is the favorable temperature for maize seed germination. All the materials had germination rates greater than 90%, evidencing a high germinative potential (Table 2). The percentage of germination ranged from 90 to 100% and heterosis ranged from − 8.5 to 3.

**Table 1:** Analysis of variance for the germination test at 25 °C in maize lines and hybrids seeds

SV	DF	SS	MS	Fc	Pr > Fc
Treatment	23	962.48	41.84	1.00	0.47^ns^
Error	65	3006.75	41.76		
CV (%) = 13.52			Mean: 95.6		

ns: not significant at 1% probability by the F test

**Table 2:** Mean values for the germination test in percentage (GER) evaluated 4, 7, 14, and 21 days after sowing and heterosis, carried out at 25 °C in maize lines and hybrids seeds

Female Parental	Male Parental	Treat	GER(%)	Heterosis	
44	44	1	99	A	–
54	54	2	96	A	–
57	57	3	94	A	–
63	63	4	96	A	–
64	64	5	98	A	–
91	91	6	99	A	–
63	44	7	99	A	1.25
64	44	8	90	A	−8.5
91	44	9	100	A	0.5
63	54	10	97	A	0.75
64	54	11	100	A	3
91	54	12	100	A	1.75
63	57	13	97	A	2.25
64	57	14	90	A	−6
91	57	15	100	A	3
44	63	16	100	A	2.25
54	63	17	96	A	0.25
57	63	18	90	A	−4.75
44	64	19	99	A	0.5
54	64	20	100	A	2.5
57	64	21	98	A	1.5
44	91	22	99	A	−0.5
54	91	23	97	A	−1
57	91	24	95	A	−1.5

Means followed by the same capital letter in the column do not differ from each other at 5% probability by the Scott Knott test

When the seeds were germinated at 10 °C, there was a statistical difference between the results of the germination percentage and the percentage of seeds with root protrusion for the evaluated materials (Table 3). This result is interesting because it reveals that the physiological quality of the seeds evaluated in the germination test was similar under favorable conditions. However, with the reduction of temperature, there was a significant reduction of the germination results of the evaluated materials.

**Table 3:** Summary of the analysis of variance for the germination (GER) and protrusion (PROT) tests carried out at 10 °C in maize lines and hybrids seeds

SV	DF	MS	
		GER	PROT
Genotypes	23	4546.6^a^	221.52^a^
Error	65	133.2	3.99
Total	88		
General Mean		39.04	94.88
CV (%)		29.56	2.10

^a^ Significant at 1% by the F test

It is worth mentioning that in the present study, only those seeds that had at least one centimeter of aerial growth and one centimeter of root growth with at least two adventitious roots were considered fully germinated, while seeds with a radicle of at least 0.5 cm were considered protruded.

For the protrusion results (Table 4), line 91 showed the highest percentage of protrusion, followed by lines 44, 57 and 64. Line 54 presented the lowest percentage of seeds with protrusion among the evaluated lines. The heterosis for protrusion ranged from − 21.25 to 10.75.

**Table 4:** Mean values in percentage for germination (GER) and protrusion (PROT) tests evaluated 4, 7, 14, and 21 days after sowing and heterosis, carried out at 10 °C in maize lines and hybrids seeds

Female Parental	Male Parental	Treat	PROT		Heterosis	GER		Heterosis
44	44	1	97	B	–	0	C	–
54	54	2	79	D	–	0	C	–
57	57	3	96	B	–	3	C	–
63	63	4	90	C	–	3	C	–
64	64	5	97	B	–	25	C	–
91	91	6	100	A	–	16	C	–
63	44	7	100	A	6.75	68	A	66.25
64	44	8	99	A	2	17	C	4.25
91	44	9	95	B	−3.75	81	A	73.25
63	54	10	63	E	−21.25	0	C	−1.25
64	54	11	95	B	7	15	C	2.75
91	54	12	100	A	10.25	46	B	37.75
63	57	13	96	B	3.25	21	C	17.75
64	57	14	96	B	−0.5	28	C	14.25
91	57	15	99	A	1.25	88	A	78.75
44	63	16	99	A	5.75	33	C	31.25
54	63	17	95	B	10.75	83	A	81.25
57	63	18	90	C	−2.75	18	C	14.75
44	64	19	100	A	3	74	A	61.25
54	64	20	100	A	12	48	B	35.25
57	64	21	95	B	−1.5	81	A	66.75
44	91	22	100	A	1.25	60	B	51.75
54	91	23	97	A	7.75	81	A	73.25
57	91	24	99	A	0.75	88	A	78.25

Means followed by the same capital letter in the column do not differ from each other at 5% probability by the Scott Knott test

For the 63/54 cross and its reciprocal 54/63, there is a 32 point variation between the heterosis of these crosses. When line 54 was used as a female parent, the protrusion result was higher than that observed when line 63 was used as a female parent. Although line 63 was introduced in the partial diallel in the tolerant to the low-temperature group, it can be observed that it showed inferior results of protrusion and germination in relation to lines 64 and 91, which can be explained by the complexity of this trait.

In the seeds of lines 44 and 54, although a high percentage of protrusion was observed, seedlings with the standard criteria established after 21 days of moistening were not observed. In the hybrids which line 44 was used as a female parent despite a high percentage of protrusion, the same behavior was not observed in relation to the germination percentage.

A heterotic effect for germination was observed for all the analyzed crosses, except the 63/54 hybrid, in which there was no statistical difference in the results observed for the germination test. The heterosis ranged from − 1.25 to 81.25, showing that hybrid vigor can and should be exploited for the production of low-temperature tolerant hybrids during the germination process.

As for the protrusion percentage results, the highest heterotic effect (10.75) was found in the 54/63 hybrid. However in the reciprocal 63/54 cross, no heterotic effect was observed (− 21.25). These results reinforce that for the hybrid combination between parents 63 and 54, line 54 should be used as a female parent so that F_1_ seeds with a better cold tolerance can be obtained. In a general evaluation, higher heterotic values were observed when crosses involved lines 54 and 91, regardless of whether they were used as a male or female parent.

### Combining abilities analysis

The mean squares for the general (GCA) and specific (SCA) combining abilities and its reciprocals effects (RE) are shown in Table 5. The results for the analysis of variance showed a significant effect at a 1% probability level for the percentage of seeds with root protrusion and percentage of germination at 10 °C.

**Table 5:** Mean squares of the general (GCA) and specific (SCA) combining abilities and reciprocal effects (RE) for protrusion percentage (PROT) and percentage of germination (TG) in maize seeds

SV	DF	MS	
		GER	PROT
GCA	5	3650.89^a^	498.96^a^
SCA	9	5821.98^a^	163.79^a^
RE	9	7850.26^a^	285.45^a^
Error	65	133.21	3.98

^a^ Significant at 1% by the F test

GCA refers to the average performance of a parent in the hybrid combinations and its significance in the analysis of variance refers to the existence of variability between the effects of GCA (Gi) associated with the additive genes. Significance for SCA (Sjj) refers to non-additive effects since SCA is an estimate of the deviations of the behavior of a hybrid in relation to that expected based on GCA.

The significance of the mean squares of the reciprocal effects (RE) indicates the existence of significant differences between the hybrids and their respective reciprocals. By examining the mean squares of the GCA and SCA effects for the germination test, the effect of SCA was found to be higher than the GCA, showing the importance of non-additive effects in controlling this trait.

In the present research, for the protrusion percentage, the effect of the GCA had a greater magnitude in relation to the SCA, evidencing the predominance of the additive effects in the expression of the trait. It can also be observed that the reciprocal effect was significant for both germination percentage and root protrusion at 10 °C (Table 4).

### Estimates of general (GCA) and specific (SCA) combining abilities and reciprocal effect (RE)

The effects of the GCA (Gi) varied for the percentage of root protrusion and percentage of germination, which reveals the differential performance of the hybrid combinations for cold stress tolerance (Table 6). For the root protrusion, the highest positive GCA estimates were observed for lines 44, 64 and 91, which indicates that these parents were better than the others evaluated when used in hybrid combinations.

**Table 6:** Estimates of the general combining ability (G), specific combining ability (S), maternal (M) and non-maternal (N) reciprocal effects for protrusion (PROT) and germination percentage (GER) when germinated at 10 °C

Parameter	PROT	GER
G1_44_	2.392	3.659
G2_54_	−5.408	−9.591
G3_57_	0.058	−1.611
G4_63_	−3.208	−7.140
G5_64_	2.192	−2.964
G6_91_	3.975	17.646
S14	5.442	14.440
S15	−0.458	16.164
S16	−1.825	15.754
S24	−8.758	18.940
S25	5.342	−18.986
S26	4.808	10.404
S34	1.775	−11.290
S35	−2.125	11.434
S36	−0.158	32.674
M1	0.838	3.533
M2	−5.384	−13.783
M3	1.949	−5.650
M4	3.968	2.117
M5	−0.032	16.867
M6	−1.338	−3.083
N14	3.630	16.083
N15	−1.370	−26.067
N16	−2.259	9.983
N24	−8.148	−25.350
N25	2.852	38.150
N26	5.296	−12.800
N34	4.519	9.267
N35	−1.481	−12.083
N36	−3.037	2.817

The numbers 1, 2, 3, 4, 5 and 6, following the letters, correspond to lines 44, 54, 57, 63, 64 and 91 respectively

More negative values were observed for lines 54 and 63. Thus, these parents were worse than the others evaluated, contributing to low performance in hybrid combinations under low-temperature conditions. However, for line 57, in spite of the GCA’s positive estimate, the estimated value was low in relation to the other lines, which indicates that the mean root protrusion of the hybrids seeds in which line 57 was used as the parent, does not differ from the average of the diallel.

In general, line 91 was the one that most contributed to increasing the percentage of root protrusion in the hybrids, whereas line 54 did not contribute in an effective way to increase tolerance to low temperatures. It is important to note that the lowest heterotic value was observed when line 54 was used as a male parent in combination with line 63 (Table 4).

Regarding the GCA in the germination percentage test, the highest positive GCA estimates were observed for lines 44 and 91 and line 91 had the highest positive estimate. Line 91 also showed a positive GCA for the protrusion percentage test. Thus, it is possible to affirm that 91 was the line with better performance in hybrid combinations for cold tolerance when evaluated through the percentages of germination and root protrusion at 10 °C. Thus, it is inferred that this line contains a higher concentration of favorable alleles for tolerance to low temperatures during the germination process and that it can be exploited in breeding programs for cold tolerance. In lines 54, 57, 63 and 64, negative estimates for GCA were observed, and the lowest one was line 54, which contributes to a lower performance of hybrids in which it participates.

The SCA (Sij) effect is the deviation of certain hybrid combinations that are relatively higher or lower than would be expected based on the GCA of their parent lines and is associated with the dominance effects of the genes, and epistasis involving dominance. The higher the value, the more divergent the combinations are, although they are also influenced by the average gene frequency of the diallel [9]. For the protrusion percentage test, no expressive results of the SCA were observed for all hybrid combinations tested. It is worth noting that for the protrusion percentage, with the exception of line 54, the lines presented high values which made the heterosis not so expressive.

Crosses between the lines 44 × 63 resulted in the best results of SCA in relation to the other crosses for protrusion. For the germination test, the hybrid 57 × 91 was the one with the highest Sij estimates. This hybrid presented 78.25% of heterosis, showing the contribution of the dominance genes in the control of this trait.

### Gene expression

Figure S1 shows the expression of the antioxidant enzymes catalase (CAT) in lines 54, 64 and in the hybrid 64 × 54 and its reciprocal 54 × 64. It is worth mentioning that for these crosses there was a RE and when it was unfolded, a maternal effect was evidenced for the crosses made with the parental 54 and 64, considering seed germination at 10 °C. In the present work, the 54 × 64 hybrid produced seeds with higher low temperature tolerance than the reciprocal cross. Despite the fact that line 64 was considered tolerant to low temperatures during the germination process, it did not behave as a good female parent when crossed with line 54. Dried seeds of Line 54 showed less catalase activity than dried seeds of Line 64 (Fig. S1).

The expression of CAT in hybrid seeds was greater in those from the 64 × 54 cross, probably due to the greater expression in the seeds of line 64. However, when the seeds were placed to germinate at 10 °C, an inversion in the expression levels of the CAT enzyme in the seeds of the evaluated materials was observed, that is line 64 shows less enzyme activity than line 54 and the hybrids. The expression was similar in hybrid seeds. It is also worth noting that, independent of the genotype, the highest expression of the enzyme was observed in seeds submitted to 10 °C for a period of seven days. This is probably a response to the stress condition, as it is known that cold stress is higher in seeds with higher water content.

**Fig. 1:**
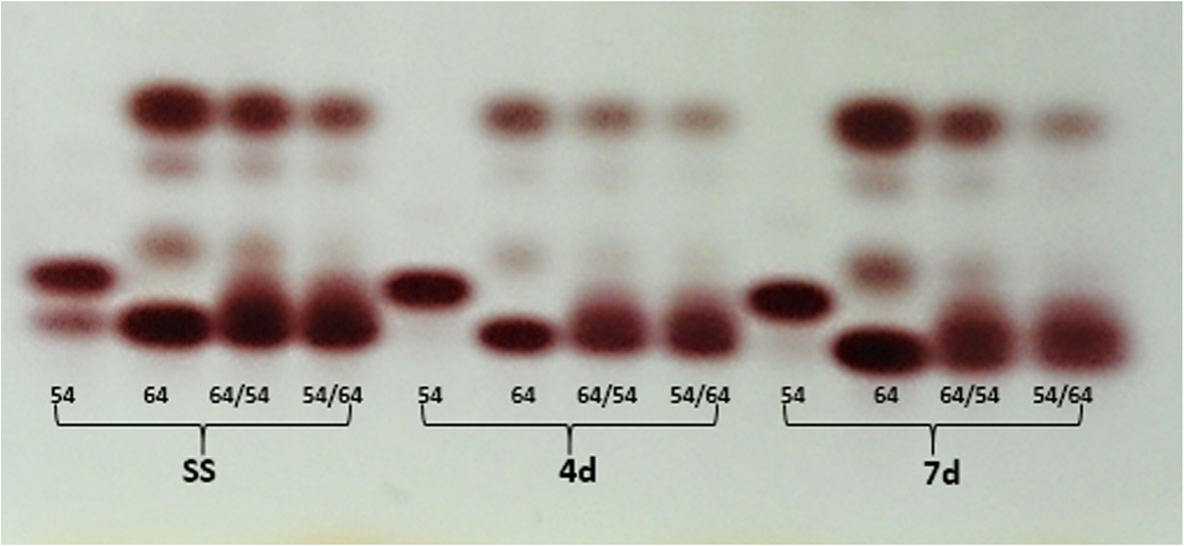
Isoenzymatic patterns of the esterase enzyme (EST) in dry maize seeds (SS) and moistened at 10 °C for 4 (4d) and 7 (7d) days in lines 54, 64 and in the hybrid 64 × 54 and its reciprocal 54 × 64

A high level of esterase (EST) activity was observed in seeds exposed to low germination temperature for seven days and in dried seeds (SS) (Fig. 1). In seeds of line 64 was observed greater expression than in seeds of line 54 and consequently, in hybrid seeds of the 64 × 54 combination, there was also a greater expression of the EST in relation to the 54 × 64 hybrid, as shown in the upper band in Fig. 1. Also, greater expression of this enzyme was observed in seeds of line 64 in relation to that observed in hybrid seeds (64/54 and 54/64), when the seeds were germinated for 7 days at 10 °C. This may be associated with lower peroxidation in hybrid seeds since EST is associated with the oxidation of membrane esters.

**Fig. 2:**
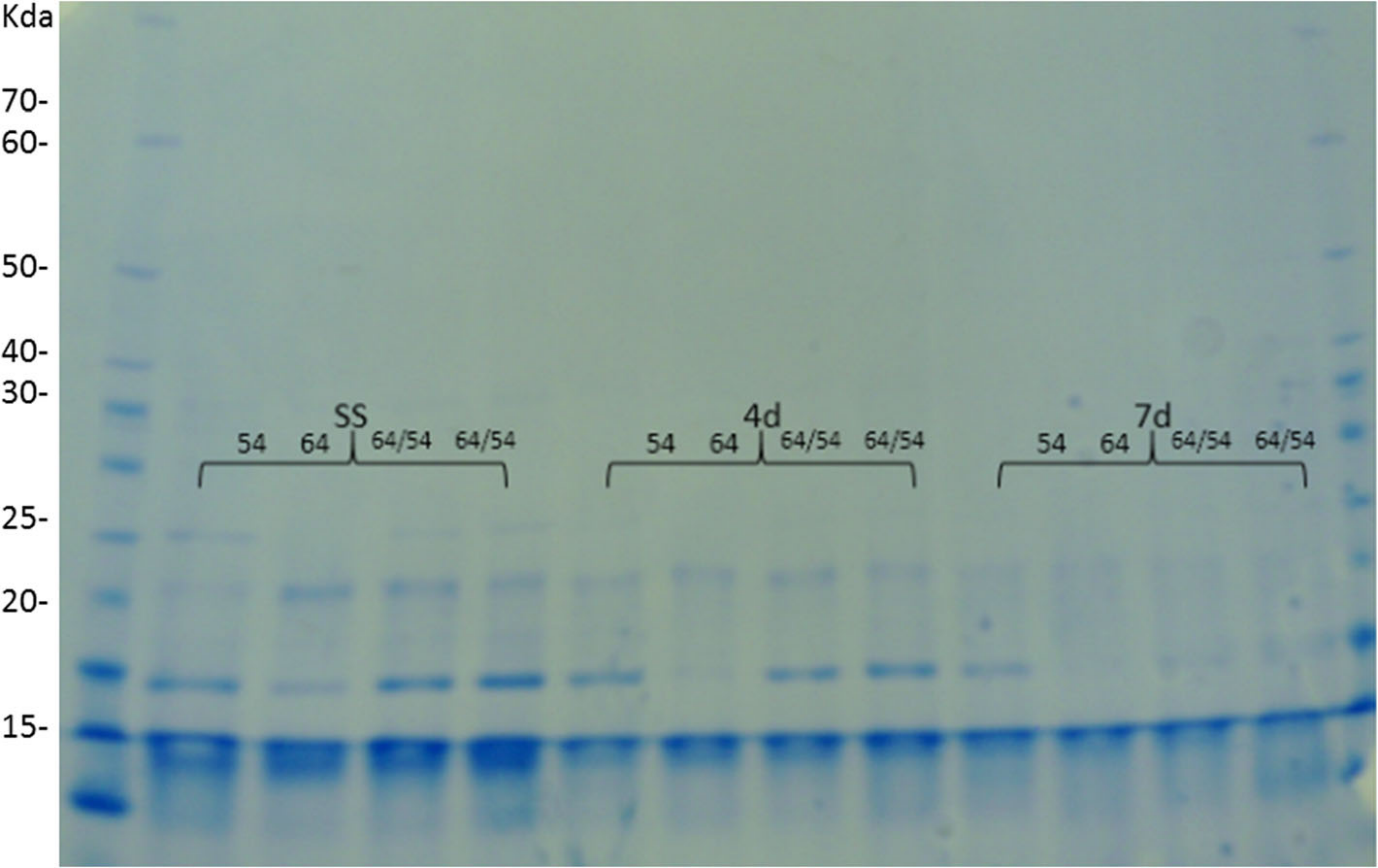
Isoenzymatic patterns of heat-resistant proteins in dry maize seeds (SS) and moistened at 10 °C for 4 (4d) and 7 days (7d) in lines 54, 64 and in the hybrid 64 × 54 and its reciprocal 54 × 64

In Fig. 2, the expression analysis of heat-resistant proteins is shown. Similar patterns were observed in dried seeds of parental lines and hybrids. However, under stress conditions, there was less expression in the seeds of line 64 in relation to the other materials. In seeds from hybrids and their reciprocal crosses, a similar level of expression was observed, unlike that observed for the expression of CAT and EST enzymes. For the heat-resistant proteins, the expression in hybrid seeds was different from seeds of the female parent. Under the conditions studied, the expression of the heat-resistant proteins in hybrid seeds was similar to the expression observed in the seeds of parental 54. Only line 64 showed lower expression as seen in the band close to 17 Kda. It is important to note that at seven days of moistening at 10 °C, there were band disappearances (molecular weights 20 and 17 Kda). It is known that with the advance of the moistening process there is a loss of tolerance to desiccation. In addition, this could also be associated with loss of tolerance to cold stress.

**Fig. 3:**
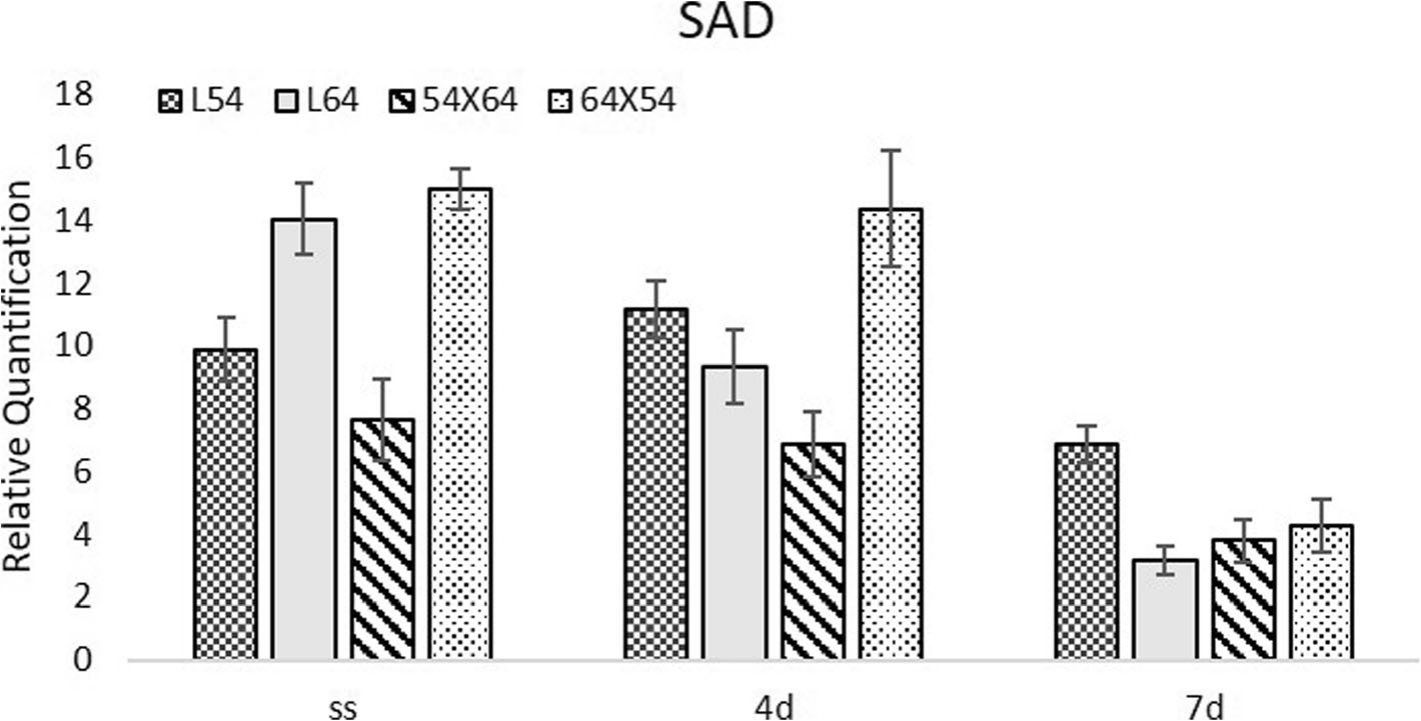
Relative expression of the SAD (Putative stearoyl-ACP desaturase) gene in dried (SS) and moistened maize seeds at 10 °C for 4 (4d) and 7 (7d) days in lines 54, 64 and in the hybrid 64 × 54 and its reciprocal 54 × 64

In general, the expression of the putative stearoyl-ACP desaturase (SAD), which acts on the establishment of fatty acids, was lower in seeds moistened at 10 °C for a period of seven days, indicating that in cold stress SAD activity was reduced for all evaluated genotypes (Fig. 3). No significant difference in SAD expression was observed in dry seeds and in those submitted to 10 °C for a period of four days. However, at 7 days this difference was very significant.

**Fig. 4:**
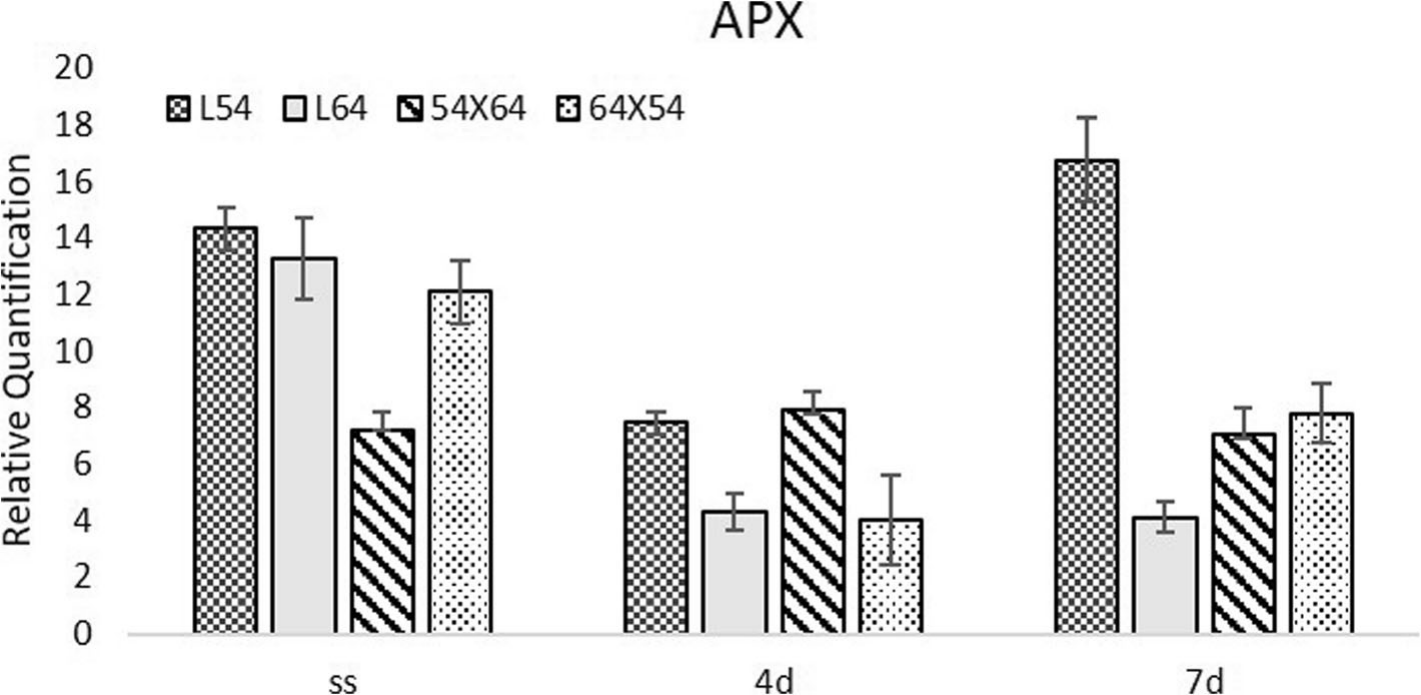
Expression of the APX (Ascorbate Peroxidase) gene in dry maize seeds (SS) and moistened at 10 °C for 4 (4d) and 7 (7d) days in lines 54, 64 and in the hybrid 64 × 54 and its reciprocal 54 × 64

In dry seeds, the activity of APX was higher in the parental lines than in the hybrids (Fig. 4). After seven days of moistening, seeds of line 54 presented the highest expression of APX in relation to the other genotypes. It can be inferred that this material was producing a greater amount of peroxide which demands more production of APX. On the fourth day of moistening at 10 °C, there was a reduction in the expression of the APX gene in the seeds of lines 54, 64 and the hybrid 64 × 54. In seeds of the 54/64 hybrid, there was no significant difference in the expression of this gene in dry seeds and in those moistened for four days at 10 °C. At seven days, the expression of this gene increased in seeds of line 54 and the hybrid 64 × 54 relative to their expression levels on the fourth day cold treatment.

**Fig. 5:**
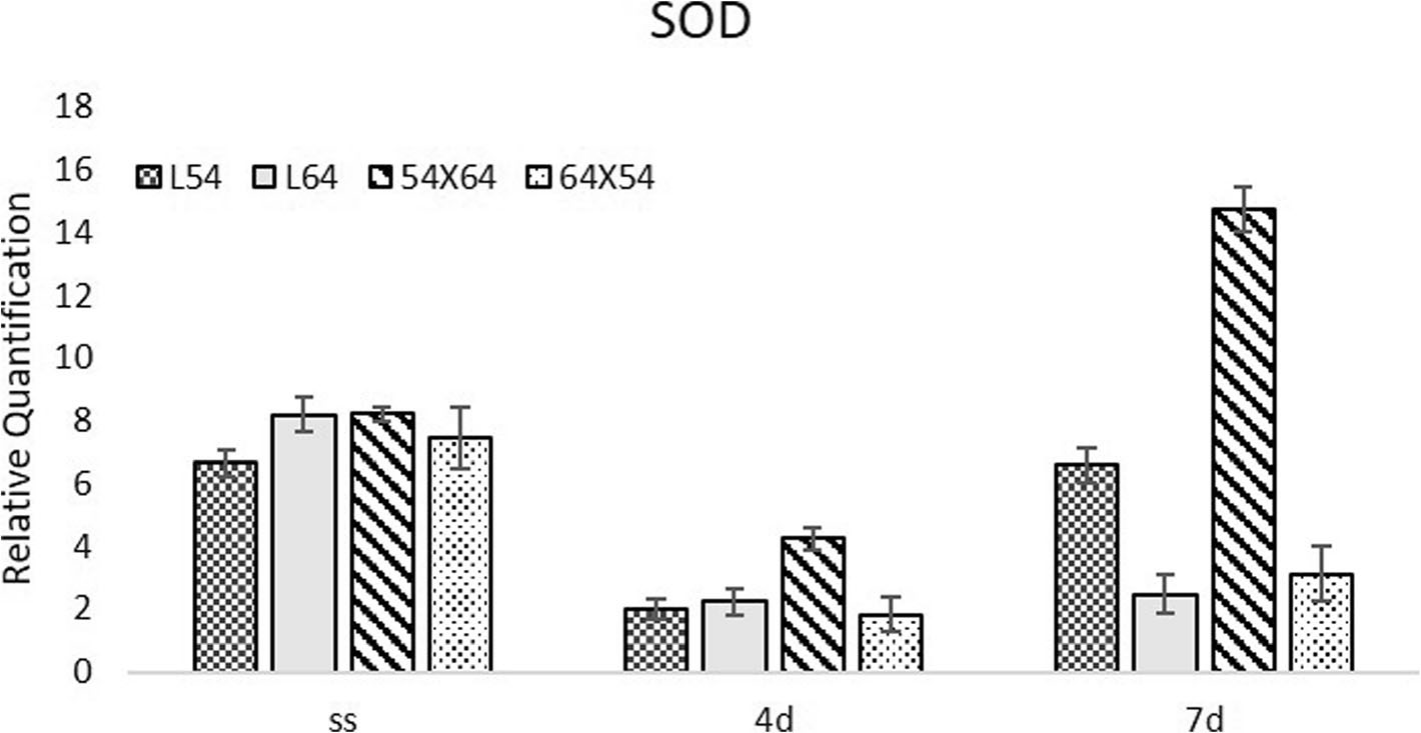
Expression of the SOD (Superoxide Dismutase) gene in dried maize seeds (SS) and moistened at 10 °C for 4 (4d) and 7 (7d) days in lines 54, 64 and in the hybrid 64 × 54 and its reciprocal 54 × 64

The level of expression of SOD showed little variation in dried seeds of the four genotypes (Fig. 5). However, under low temperature stress for four days, a higher level of SOD expression was observed in the seeds of the 54 × 64 hybrid, and this level of expression increased even more after 7 days and was greater than all other genotypes. In our analysis, the best germination percentage was observed in 54 × 64 hybrid cross when compared to the other genotypes analyzed. In dry seeds, there was less SOD expression for line 54. On the fourth day of moistening at 10 °C, there was a significant reduction of SOD expression in seeds of all genotypes studied. At four and seven days cold treatment, higher levels of expression were observed in seeds of the 54 × 64 hybrid.

**Fig. 6:**
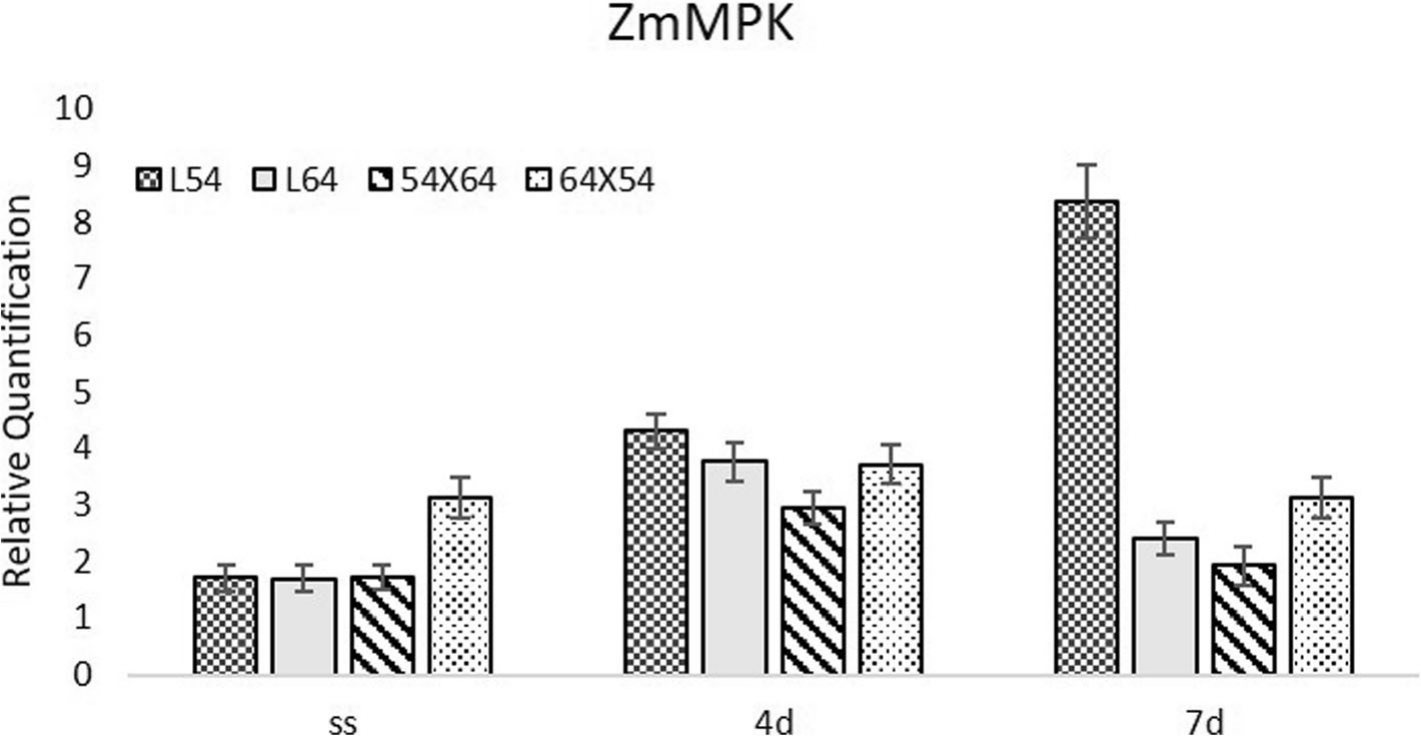
Expression of the *ZmMPK5* (Mitogen Activated Protein Kinase) gene in dry maize seeds (SS) and moistened at 10 °C for 4 (4d) and 7 (7d) days in lines 54, 64 and in the hybrid 64 × 54 and its reciprocal 54 × 64

In Fig. 6, the expression of the *ZmMPK5* (Mitogen Activated Protein Kinase) gene is shown. The accumulation of ABA and hydrogen peroxide contributes to a higher expression of this gene [10]. *ZmMPK5* expression was higher in seeds moistened at 10 °C for 4 and 7 days than in dry seeds for lines 54 and 64. For the 54 × 64 hybrid, a higher expression was observed only at four days. Higher expression of this gene was verified in seeds moistened for 7 days at 10 °C in seeds of line 54. This line exhibited a lower percentage of root protrusion in relation to the other lines. Also, seedlings with the minimum standard established in this research were not observed for line 54. This intolerance to low temperature may be due to the accumulation of ROS and also to the higher content of abscisic acid, since these two factors may have contributed to a greater expression of *ZmMPK5*.

## Discussion

The germination results of the present study agree with the ones obtained by Farooq et al. [11], who verified that low temperature had a significant effect on the germination and growth traits of maize hybrid seedlings. In the study conducted by Farooq et al. [11], low temperatures increased the time for the seedlings to emerge, increased the expression of the superoxide dismutase enzyme, reduced the emergence percentage, the emergency uniformity coefficient and the root length.

Similar results were also found in an experiment with three temperature conditions and thirty different hybrids, in which significant effect of the genotypes was verified in different temperature conditions and it was found that under low-temperature conditions the root system and the aerial part of the plant were less developed because of the cold stress, which significantly reduces cell division and elongation [12].

According to the results for heterosis in the hybrids and the reciprocal crosses, we can conclude that there is a heterotic and reciprocal effect for cold tolerance during the germination process. These results further reinforce the importance of analyzing the best hybrid combinations, not only aiming for better final productivity but also the high physiological quality of the seeds, since seeds are the first input for establishing the crop in the field.

The results obtained in the 63/54 cross and its reciprocal 54/63 crossing do not corroborate with those found by Kocová et al. [13]. The authors stated that in order to obtain better cold tolerance in hybrids, the more tolerant materials should always be used as the female parental. In crosses 57/63 and 57/64, a negative heterotic effect is observed, suggesting that line 57 is not a good female parent for protrusion under low-temperature conditions.

For a cultivar to have good cold stress tolerance, it is very important that the seeds have fast germination, and originate normal seedlings even in the low-temperature condition. In this sense, the evaluation of protrusion percentage is important to verify that the seeds have the germinative process initiated even in low-temperature conditions, and it is essential that the seedlings develop rapidly and uniformly. What can be observed in Table 4 is that in many materials with high percentage values of root protrusion, no significant values for germination were observed.

The permanence of the protruded seeds in the soil can favor the attack of pests and diseases. In addition, the longer the seedling takes to emerge, the less competitive it will be in relation to weeds. The use of low vigor seeds is associated with an increase in weed biomass of 169–210% and a decrease in crop productivity of 16–21% [14]. So, it is necessary for the seedling to have the genetic potential to withstand low temperatures.

In the germination test, although many lines had a high percentage of protruded seeds, these lines were often not considered to be fully germinated (Table 4). In order to tolerate low-temperature conditions, it is necessary to establish a fast and uniform plant stand, so it is essential not only to evaluate the ability of the seeds to start the germination process but also to evaluate the ability of seeds to produce normal seedlings even under cold conditions. In general, the results of the germination of the hybrids were higher than those observed in seeds of the lines (Table 4). These results are in accordance with several studies in which heterosis was observed in germination and seed vigor [15–19].

These results corroborate Meyer et al. [20], which stated that during the early stages of embryo development, there is greater gene expression and higher metabolic activity in the seeds of the hybrids in relation to that observed in the parents, which provides greater metabolic efficiency and, therefore, better performance of the hybrid.

Kollipara et al. [21] crossed contrasting lines for cold tolerance during germination and observed high heterotic values in the hybrids generated. The authors also observed, through the cold test, reciprocal effects among hybrids, and the hybrid with the highest germination value was the one with the highest quality pedigree in this test, showing the importance to correctly choose the female genitor to obtain seeds with tolerance to low temperatures in the germination stage. However, in the present study, a direct correlation between greater tolerance to cold stress and the use of more tolerant lines as a female parent was not identified.

The major importance of non-additive effects on the physiological quality of seeds was also reported by Cabral et al. [22] when evaluating seeds of ten popcorn lines in a full reciprocal diallel cross. Similar results were reported by Gomes et al. [15] for the physiological quality of tropical maize seeds, in which the authors concluded that there were significant effects for both GCA and SCA and that the non-additive effects were more significant for seedling emergence, germination speed index, length of aerial part and root of seedlings, speed of emergence, among others.

In cold tolerance during the germination process in maize cultivars, the RE proved to be significant, and the superiority of the tolerant x susceptible to the susceptible x tolerant crosses was significantly. An important contribution of this reciprocal effect is due to the maternal effect. It is interesting to note that the maternal effect for cold tolerance is related to the capacity per se of the parental line, indicating that the greater the effect per se, the greater is the maternal effect [23].

According to Cockerham and Weir [24], the RE can be unfolded in maternal and non-maternal effects. This unfolding allows inferring the genetic causes of the RE. The maternal-effect is caused by cytoplasmic genes or the interaction between cytoplasmic and nuclear DNA, so the trait is inheritable and can be exploited in breeding programs, while the non-maternal effect is directly related to the environment effect. The occurrence of maternal effect should always be considered during the choice of hybrid crosses, once it has been demonstrated that there is an effect of the female parental on the seed quality. The unfolding of the RE is of fundamental importance for understanding the genetic control of cold tolerance during the germination process, allowing the breeder to make more assertive decisions regarding this trait.

Cold tolerance is a complex trait that is associated with several factors such as physiological quality, biochemical characteristics, and expression of antioxidant enzymes. These enzymes are important for the removal of reactive oxygen species (ROS). In higher plants, ROS are generated during normal aerobic metabolisms, such as photosynthesis and respiration. However, higher levels of ROS can be produced under stress conditions, such as low temperatures, which can result in damage to DNA, proteins, and lipids. Under biotic and abiotic stresses, antioxidant enzymes play an important role in the removal of this ROS [25].

CAT is an antioxidant enzyme that catalyzes the conversion of hydrogen peroxide into water, protecting cells from oxidation caused by free radicals. The faster the metabolism of the seed, the greater the production of ROS and this production is accentuated in a stress condition. Similar expression of CAT in the hybrids evidence that there is a heterotic effect for the expression of this enzyme when the maize seeds are moistened under low temperatures. EST participates in ester hydrolysis reactions and can act on membrane phospholipids in seeds, causing lipid peroxidation. It is worth mentioning that the stability of the membrane is one of the fundamental factors for seed tolerance at low germination temperature.

According to Menezes et al. [26], heat-resistant protein patterns are polymorphic and stable in maize seeds with different levels of quality, which makes this class of proteins an excellent marker in the identification of cultivars. For Fu et al. [27], heat-resistant proteins between 12 and 40 KDa act as protectors during germination. These authors also suggest that these proteins are related to the appearance of heterosis during germination. The greater expression of heat-tolerant proteins for line 54 relatively to line 64 could have contributed to the better performance of the hybrids when line 54 was used as a female parent.

The mechanisms involved in cold tolerance have also been studied through the expression of transcripts in a range of species, including *Arabidopsis thaliana* [28], wheat [29] and barley [30]. Real-time PCR enables quantification of target gene expression and thus provides a more in-depth analysis of the influence of the gene on the characteristic of interest. The results of the gene expression analysis, in general, showed variation of gene expression in the dried seeds and after 4 and 7 days of moistening at 10 °C.

SAD converts saturated fatty acid to unsaturated fatty acids, and this conversion is an important mechanism of tolerance in plants under low-temperature conditions. In *Arabidopsis thaliana*, the expression of desaturase (FAD8) was strongly induced by low temperature [31]. Liu et al. [32] studied the expression of the desaturase (*LeFAD7*) in tomato leaves and noted that it was induced by cold stress (4 °C), and inhibited by high temperature (45 °C). In rice, the *OsFAD2* gene was suggested to confer stress resistance on plants developed under temperatures not ideal for the culture [33].

The enzymatic antioxidant defense system in plants is comprised mainly of superoxide dismutase (SOD), catalase (CAT), ascorbate peroxidase (APX) and glutathione peroxidase (GPX). APX are proteins that neutralize peroxides using ascorbate, these proteins are part of the group of the main antioxidants in plant cells. When superoxides are generated as a by-product of photosynthesis or NADPH oxidation, SOD rapidly converts superoxides into relatively stable and neutral hydrogen peroxide (H_2_O_2_) molecules. APXs clean hydrogen peroxides and neutralizes them through the ascorbate-glutathione cycle [34].

Abscisic acid (ABA) is a phytohormone known to regulate the reactions of development and growth of plants in response to stresses and modulate the initiation and maintenance of seed dormancy [35]. ABA stabilizes the dormancy state of seeds to ensure that germination occurs only under adequate environmental conditions [36].

The results of the genetic control analysis revealed the presence of heterosis and RE with a large influence of non-maternal effects associated with protein and gene expression results, all of which reinforce the complexity of the cold tolerance in maize seeds during the germinative process. The important influence of non-additive effects and the existence of heterosis and RE reinforces the importance of testing the hybrid combinations, as well as the choice of which parent will be used as a female parental and which will be used as a male.

## Conclusions

It may be suggested that research focused on studies of hybrid combinations linked to biochemical and molecular analyses can be used to provide a better understanding of the mechanisms involved in tolerance to abiotic stresses and contribute to the selection and development of materials tolerant to these stresses.

The results of this study revealed heterosis for cold tolerance during the germination process in maize seeds, with a greater contribution due to the non-additive gene effects. The catalase enzyme was found to increase during the germination process, with higher levels of expression after seven days of exposure to moisture and cold temperature; while heat-resistant proteins showed the opposite effect. There was variable expression of APX, SOD, and *ZmMPK5* genes both among genotypes and in response to low temperature under moist conditions. Most importantly, the results reveal the necessity to evaluate hybrid combinations and their reciprocal crosses to determine the best hybrid combinations for improved cold tolerance during seedling establishment of the next generation.

## Methods

### Seed production

The research was conducted at the Federal University of Lavras (UFLA), in Lavras, MG, Brazil. With a latitude of 21°14′ S, 40°17′ W longitude and altitude of 918.80 m. This region presents a Cwb climate in the Koppen classification. The average annual temperature is 19.4 °C and rainfall is mainly distributed from October to April, with annual values of 1529.7 mm.

Six maize lines were used, three of which were classified as tolerant to low germination temperature (91, 64 and 63) and three were classified as not tolerant to this stress condition (44, 54 and 57). The selection of the materials was carried out according to the results obtained in the research developed by Silva-Neta et al. [1]. The plant material was provided by the Lavras Federal University maize breeding program. A field for the multiplication of the six lines and the production of the hybrids was installed in a partial diallel scheme including the reciprocal crosses. Therefore, seeds of 24 maize genotypes were produced.

The soil was conventionally prepared and fertilized based on chemical analysis. The spacing of 0.8 m between rows and seven plants per linear meter was used. Covering fertilization, as well as other cultural treatments, was carried out according to those recommended for maize cultivation. The ears were subjected to artificial drying at 35 °C until the seeds reached the water content of approximately 13%. For the evaluation of the physical and physiological quality of maize seeds, the water content was determined and germination tests were carried out at temperatures of 10 and 25 °C.

The water content was determined by the greenhouse method at 105 °C for 24 h, using two subsamples of each material, according to the Brazilian Rules for Seed Analysis - RAS [37]. The results were expressed as a mean percentage of humidity and was made to ensure that the water content of each material was close to 13%. Four replicates of 50 seeds were used in the germination test for each temperature, using the Germitest type paper moistened with distilled water in the proportion of 2.5 times the weight of the dry paper. The rolls were packed in plastic bags and kept in a B.O.D. type chamber, regulated at temperatures of 10 and 25 °C (+ or - 3 °C).

At 10 °C, it takes up to 21 days for root protrusion to be observed in some materials, so the evaluations were performed at 4, 7, 14 and 21 days after sowing. The results were expressed as the mean percentage of normal seedlings that germinated in the four replicates. Seedlings with at least 1 cm of the main root, two adventitious roots with at least 1 cm and 1 cm of aerial part (shoot) were considered fully germinated. The protrusion percentage was also calculated, counting all seedlings that had at least 0.5 cm of radicle growth.

For the germination tests at 10 °C and 25 °C, carried out to evaluate the physiological quality of the seeds, a completely randomized design with four replications was used, and each replication was done in a different growth chamber for each temperature. The data were interpreted statistically by analysis of variance and the means compared by the Scott Knott test at a 5% level. The analyses were performed in the statistical program R.

### Estimates of general and specific combining abilities and reciprocal maternal and non-maternal effects

Based on the results of the analysis of variance, the sums of squares of the treatments were decomposed into general combining ability (GCA), specific combining ability (SCA) and reciprocal effects, the latter was unfolded in maternal and non-maternal effects. Adapting the model proposed by Griffing [38], to describe the experimental observations, we have the following characterization:

$$ \mathrm{Yij}=\upmu +\mathrm{gi}+\mathrm{gj}+\mathrm{Sij}+\mathrm{Rij}+\mathrm{eij} $$

Where:

i = 1, 2, ..., p, where *p* = 3, which corresponds to group 1, lines with low physiological quality of seeds;

j = 1, 2, ..., p, where p = 3, which corresponds to group 2, lines with high physiological quality of seeds;

Yij: mean value of the hybrid combination between the i-th genitor of group 1 and the j-th genitor of group 2;

μ: overall mean;

gi: effect of the general combining ability of the i-th parent of group 1;

gj: effect of the general combining ability of the j-th parent of group 2;

Sij: effect of the specific combining ability between genders i and j, of groups 1 and 2, respectively;

Rij: reciprocal effect of crossing ij: Rij = Rji, E (Rij) = Rij; and.

Eij: mean experimental error.

All effects were assumed to be fixed in this analysis, in order to estimate the effects of the parents alone, therefore, some restrictions were imposed to estimate the GCA, SCA and reciprocal, maternal and non-maternal effects. Such as: Σgi = 0; ΣSij = 0 for each j with Sij = Sji; Rji = Rij; Σmi = 0; Σnmi. = Σnm.j = 0 with nmij = −nmji.

### Protein analysis

Seeds of lines 54 and 64 (the most contrasting lines for cold tolerance [1]) were used for the protein analysis, as well as the seeds of the hybrid combination between these and their reciprocals. The analyses were performed with dried seeds and seeds moistened at 10 °C for 4 and 7 days.

For the extraction of the heat-resistant proteins, the seeds were ground in liquid nitrogen. Then buffer (50 mM Tris-HCl-7.5, 500 mM NaCl, 5 mM MgCl 2, 1 mM PMSF) was added in the proportion of 1:10 (weight of material: volume of extraction buffer), and the supernatant was transferred to microtubes of 1500 μL capacity. The samples were centrifuged for 30 min at 14,000 rpm and 4 °C. A water bath for 15 min at 85 °C was used to incubate the supernatant and the samples were centrifuged again in the same way. The supernatant was latter poured into microtubes and the pellet discarded. Prior to application to the gel, the sample tubes containing 70 μL extract + 40 μL sample buffer (2.5 ml glycerol, 0.46 g SDS, 20 mg Bromophenol blue and the volume completed to 20 ml Tris extraction buffer, pH 7.5) were placed in a water bath with boiling water for 5 min [39]. 50 μL of the extract with protein + sample buffer was applied in each well, on a 12.5% SDS-PAGE polyacrylamide gel (separator gel) and 6% (concentrator gel). The electrophoretic run was performed at 120 V and the gels stained with Coomassie Blue at 0.05% (diluted in water) for 12 h and destained in 10% acetic acid solution [40].

For the extraction of the catalase (CAT) and esterase (EST) enzymes, the seeds were ground in liquid nitrogen and then extracted with 0.2 M Tris HCl buffer pH 8.0 containing 0.1% mercaptoethanol (100 mg tissue / 250 μL buffer). The tubes were vortexed and stored for 12 h in a refrigerator, followed by centrifugation at 14,000 rpm for 30 min at 4 °C. Proteins (60 μL of supernatant) were separated using a 4.5% (concentrator gel) and a 7.5% polyacrylamide gel (resolving gel). The electrophoresis was performed at 120 V for 5 h. The gels were stained following the protocol of Alfenas [40], to reveal the enzyme activities.

### Expression analysis by qRT-PCR

For the expression analysis, seeds of lines 54 and 64 were used, as well as seeds of the hybrid combination between these lines and their reciprocal crosses. The analyses were performed on dry seeds and seeds moistened at 10 °C for 4 and 7 days.

In the RNA extraction, the seeds were ground in liquid nitrogen and then added the Pure Link RNA Plant® reagent (Invitrogen), following the specifications of the manufacturer’s manual. After extraction of the nucleic acids, the samples were treated with DNase-free to eliminate any DNA contamination. For this, the DNAse Turbo Free® kit (AMBION) was used. To verify the effectiveness of the DNA removal, a conventional PCR reaction was performed. As a positive control, a sample of maize genomic DNA was used.

After the extraction and purification process, mRNAs were used as templates for cDNA synthesis. The cDNA Reverse Transcription cDNA® kit (Applied Biosystems) was used according to the protocol recommended by the manufacturer. The efficiency of cDNA synthesis was confirmed by standard PCR. In this analysis, the maize genomic DNA sample, using the primer corresponding to the constitutive gene Ubiquitin, was used as the positive control. A 1.5% agarose gel stained with ethidium bromide was prepared for visualization.

Sequences of the chosen target genes were found by searching the genomic database of maize sequence in GenBank (www.maizegdb.org). Based on these sequences, the primers were designed using Primer Express 3.0 software (Applied Biosystems). The sequences of the primers used are shown in Table 7. As a reference gene, the Ubiquitin gene was used.

**Table 7:** Primers used in the qRT-PCR analysis

Gene	Identification		Sequence 5′ - > 3’	GenBank Acession Number
*Ubiquitin*	Reference gene	F	AAGGCCAAGATCCAGGACAA	GRMZM2G027378_T01
		R	TTGCTTTCCAGCGAAGATGA	
*SAD*	Putative stearoyl-ACP desaturase	F	GGA TTT CCT CCC TGA CCC A	KU949326
		R	GTC CAT GCC CTC GTC CAA A	
*ZmMPK5*	Mitogen Activated Protein Kinase	F	ACTGATGGACCGCAAACC	AB016802
		R	GGGTGACG AGGAAGTTGG	
*SOD*	Superoxide Dismutase/Antioxidant	F	TGGAGCACCAGAAGATGA	X17565
		R	CTCGTGTCC ACCCTTTCC	
*APX*	Ascorbate Peroxidase/Antioxidant	F	TGAGCGACCAGGACATTG	EU969033
		R	GAGGGCTTTGTCA CTTGGT	

(F) sequence of the forward primer and (R) sequence of the reverse primer

The ABI PRISM 7500 Real-Time PCR Instrument (Applied Biosystems) was used with SYBR Green for expression analysis of the selected genes. cDNA samples obtained from dried and moistened seeds for 4 and 7 days at 10 °C were used in biological triplicates. The efficiency of the drawn primers was determined by means of an absolute quantification dilution curve. All primer efficiencies were between 97.45 and 102.71.

In the expression analysis, 1 μL of cDNA (diluted 1: 5), 0.4 μL of primer forward/reverse (10 μM) and 5 μL of Master MixSYBR green (Applied Biosystems) were used in each reaction, totaling a final volume of 10 μL. The samples were pipetted in technical triplicates, and a control without cDNA (NTC) was included for each pair of primers. The results were normalized using CTs (Threshold Cycle) obtained by expression of the reference gene Ubiquitin. The CT was determined by the number of cycles at which the fluorescence generated within a reaction crosses the threshold cycle (CT). The relative expression was analyzed by the Pfaffl method [41]. The thermal conditions of the reaction were: 2 min at 50 °C and 10 min at 95 °C for the initiation, followed by 40 cycles of 15 s at 95 °C and 1 min at 60 °C, and ending with 15 min at 95 °C. At the end of the cycles, the specificity of the PCR reaction was evaluated by means of the denaturation curve of 60–95 °C.

## Supplementary information

**Additional file 1: Figure S1.** Isoenzymatic patterns of the catalase enzyme (CAT) in dry maize seeds (SS) and moistened at 10 °C for 4 (4d) and 7 (7d) days in lines 54, 64 and in the hybrid 64 × 54 and its reciprocal 54 × 64.

## Data Availability

The datasets used and/or analyzed during the current study are available from the corresponding author on reasonable request.

## Abbreviations

QTL: Quantitative trait loci
GCA: General combining ability
SCA: Specific combining ability
CT: Threshold cycle
RE: Reciprocal effect
ROS: Reactive oxygen species
CAT: Catalase
EST: Esterase
SS: Dried seeds
SAD: Putative stearoyl-ACP desaturase
SOD: Superoxide dismutase
APX: Ascorbate peroxidase
*ZmMPK5*: Mitogen Activated Protein Kinase
ABA: Abscisic acid
