# Supplementary figures and images for: Gene expression and genetic control to cold tolerance during maize seed germination
Source: BMC Plant Biol. 2020 Apr 29;20:188. doi: 10.1186/s12870-020-02387-3 (PMC7191758; doi:10.1186/s12870-020-02387-3)

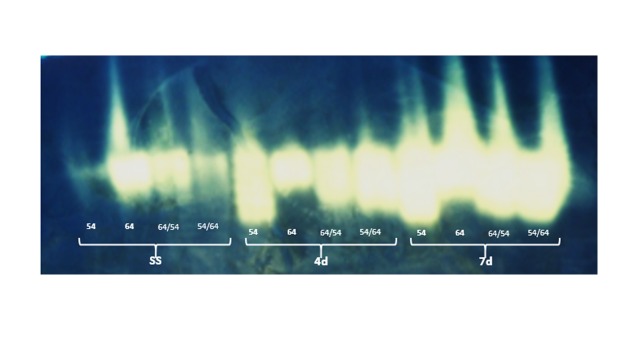

Supplement: Supplementary file 1 — Additional file 1: Figure S1. Isoenzymatic patterns of the catalase enzyme (CAT) in dry maize seeds (SS) and moistened at 10 °C for 4 (4d) and 7 (7d) days in lines 54, 64 and in the hybrid 64 × 54 and its reciprocal 54 × 64. [file 12870_2020_2387_MOESM1_ESM.tif]
